# Recent Advances in Fluorescent Polyimides

**DOI:** 10.3390/molecules29174072

**Published:** 2024-08-28

**Authors:** Manyu Lian, Liyong Tian, Guotao Huang, Siming Liang, Yangfan Zhang, Ningbo Yi, Longfei Fan, Qinghua Wu, Feng Gan, Yancheng Wu

**Affiliations:** College of Textile Science and Engineering, Wuyi University, Jiangmen 529020, China

**Keywords:** fluorescent polyimide, fluorescence, CT interactions, structure

## Abstract

Polyimide (PI) refers to a type of high-performance polymer containing imide rings in the main chain, which has been widely used in fields of aerospace, microelectronic and photonic devices, gas separation technology, and so on. However, traditional aromatic PIs are, in general, the inefficient fluorescence or even no fluorescence, due to the strong inter- and intramolecular charge transfer (CT) interactions causing unavoidable fluorescence quenching, which greatly restricts their applications as light-emitting functional layers in the fabrication of organic light-emitting diode (OLED) devices. As such, the development of fluorescent PIs with high fluorescence quantum efficiency for their application fields in the OLED is an important research direction in the near future. In this review, we provide a comprehensive overview of fluorescent PIs as well as the methods to improve the fluorescence quantum efficiency of PIs. It is anticipated that this review will serve as a valuable reference and offer guidance for the design and development of fluorescent PIs with high fluorescence quantum efficiency, ultimately fostering further progress in OLED research.

## 1. Introduction

In recent years, luminescent polymeric materials with high photoluminescence quantum yield (*Φ*_PL_) have been successfully applied in the fields of polymer light-emitting diodes (PLEDs) [1,2], optical wavelength converters [3], plastic lasers [4], fluorescent sensors [5], and so on, due to their additional advantages of flexible structure, low cost, and easy film-forming compared with organic small-molecule luminescent materials [6]. There are many luminescent polymeric materials, such as polyacetylene [7], polythiophene [8], polyaniline [9], polyamidoamine [10], and polyamino-ester [11], that have been reported. However, these luminescent polymeric materials have some shortcomings of complex polymerization and purification processes, poor thermal and dimensional stabilities, and bad mechanical strength of nanoscale thin films, which restrict the in-depth application of the luminescent polymeric materials in modern optical and microelectronic technology. For example, the processing temperature for the organic light-emitting diode (OLED) devices could be over 300 °C, which requires that the luminescent polymeric materials for constructing high-performance OLED devices simultaneously possess excellent heat resistance with high glass transition temperature (*T*_g_) and low coefficient of thermal expansion (CTE) [12]. Therefore, most conventional luminescent polymeric materials have difficulty meeting the requirements of high processing temperature in the fabrication of OLED devices.

Polyimides (PIs) refer to a type of high-performance polymers containing imide rings in the main chain, which have excellent thermal and dimensional stabilities, and good mechanical properties [13,14]. They have been widely used in fields of aerospace, microelectronic and photonic devices, gas separation technology, and so on [15,16,17]. With regard to OLED applications, PI materials with excellent thermal stability are mostly used as the transport layer, device substrate, or luminescent layer doped with a luminescent material [18,19,20,21]. Only a few cases have been reported in which fluorescent PI has been directly used as the luminescent layer in the fabrication of OLED devices [1,22,23]. This is because the conventional aromatic PIs show the inefficient fluorescence or even no fluorescence, due to the strong inter- and intramolecular charge transfer (CT) interactions and the lowest excited CT (π–π*) states causing unavoidable fluorescence quenching [24,25,26]. If the PI materials with excellent heat resistance have high fluorescence quantum efficiency, they are very suitable for use as the luminescent layer in the fabrication of OLED devices. Therefore, it is important to develop fluorescent PIs with high fluorescence quantum efficiency.

At present, the main synthetic strategies to improve the fluorescence quantum efficiency of PIs are suppressing CT interactions in PIs through molecular structure design. Basing on this idea, two methods have been proposed and studied to improve the fluorescence quantum efficiency of PI, including introduction of aliphatic/alicyclic segments into the PI main chain and the specific pendant side functional groups into the aromatic PI chain. In this paper, the research progress of fluorescent PIs in recent years is systematically reviewed, and various strategies to improve the luminescent performance of PIs are summarized. Further, the application of fluorescent PI materials is discussed, and the future research direction of high-performance and efficient fluorescent PI materials is prospected.

## 2. Fluorescent Semiaromatic PIs Containing Alicyclic Diamine Segments

The CT interactions in semiaromatic PIs are effectively suppressed by introducing alicyclic diamines, regardless of the dianhydride structure, due to the nonaromatic nature and weak electron-donating property of alicyclic diamines [27,28]. The lowest excited states of semiaromatic PIs derived from aromatic dianhydrides having delocalized π conjugation and an alicyclic diamine are attributable to localized excitation (LE) (π–π*) states which could emit strong fluorescence. Therefore, the fluorescent semiaromatic PI is easily obtained by using the aliphatic/alicyclic diamines and aromatic dianhydrides [29,30,31].

**Figure 1:**
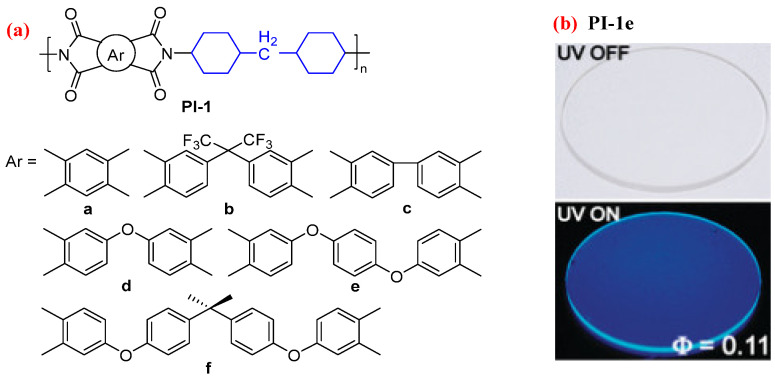
(**a**) The chemical structures of PI-1. (**b**) Photos of PI-1e films under sunlight and 365 nm UV light. Adapted with permission from Ref. [27]. Copyright 2009, ACS Publications.

For example, some fluorescent semiaromatic PIs (PI-1, Figure 1a) were prepared from 4,4′-diaminocyclohexylmethane (DCHM) and aromatic dianhydrides [27]. The lowest excited states of PI-1 derived from aromatic dianhydrides having delocalized π conjugation and an alicyclic diamine are attributable to LE (π–π*) states, which emit strong fluorescence. In particular, PI-1e having two ether (-O-) linkages in the dianhydride moiety showed the strongest blue fluorescence, with a highest absolute *Φ*_PL_ value of 0.11 (Figure 1b). This because the bent and rotatable diphenyl ether structure prevents dense chain packing, which effectively weakens the fluorescence. However, the lowest excited states of PI-1a and PI-1b derived from dianhydrides with localized π conjugation and an alicyclic diamine are attributable to LE (n-π*) states, which emit quite weak fluorescence with the lower *Φ*_PL_ values of <0.03.

**Figure 2:**
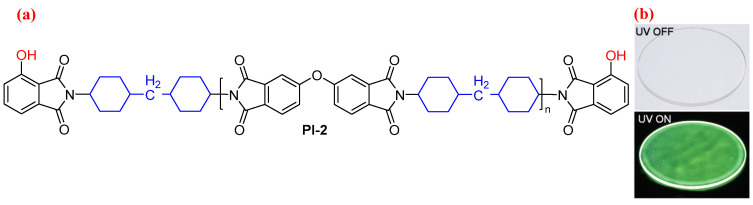
(**a**) The chemical structures of PI-2. (**b**) The photos of PI-2 (r = 7.69) under white light and 365 nm UV light. Adapted with permission from Ref. [32]. Copyright 2010, ACS Publications.

Excited state intramolecular proton transfer (ESIPT) is one of the distinctive photophysical processes of fluorescent compounds and polymers, which induces enhanced fluorescence [32,33]. In this regard, Ando et al. [32] first developed a series of highly fluorescent PIs, prepared from ODPA and DCHM end-capped with 3-hydroxyphthalic anhydride (PI-2, Figure 2a). Due to the presence of ESIPT fluorophore of 3-hydroxyphthalic anhydride, these PIs showed gradated multicolor ESIPT emission (blue, light-blue, white, and light-green) depending on the amount of fluorescent termini with *Φ*_PL_ values of 9–14% (e.g., PI-2, the molar ratio of end group (r) is 7.69, Figure 2b). It was clarified that emission is a useful tool for controlling the fluorescence properties of PIs.

**Figure 3:**
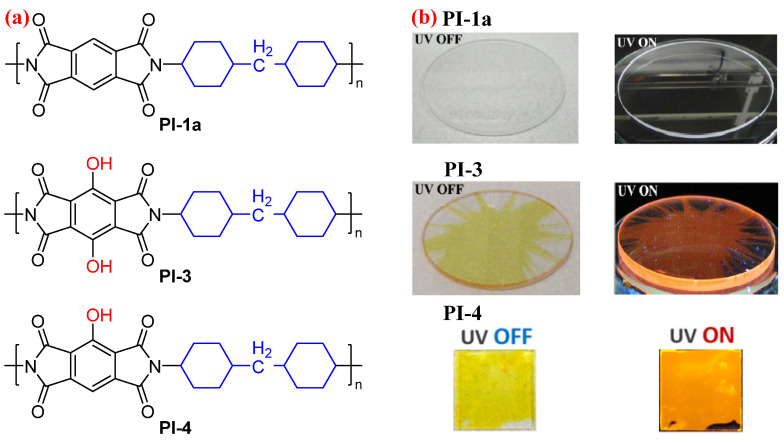
(**a**) The chemical structures of PI-1a, PI-3, and PI-4. (**b**) The photos of PI-1a, PI-3, and PI-4 films under sunlight and 365 nm UV light. Adapted with permission from Refs. [34,35]. Copyright 2015 and 2016, ACS Publications, respectively.

Later, Ando et al. [34] further designed a semiaromatic PI containing hydroxyl groups in the PMDA-based dianhydride moiety (PI-3, Figure 3a), which exhibits fluorescence originating from the ESIPT process. Compared with the very weak fluorescent PMDA-based PI film (PI-1a, Figure 3b) without two hydroxyl groups in the dianhydride moiety with a *Φ*_PL_ value of 0.004 [27], the PI-3 film containing two hydroxyl groups exhibited prominent red emission at 642 nm with a very large Stokes shift of 8994 cm^−1^ and a *Φ*_PL_ value of 0.01 via ESIPT [34]. The still relatively low *Φ*_PL_ value of PI-3 film is due to its planar structure of hydroxyl-containing PMDA-based dianhydride moiety, which is highly likely to form the aggregates via hydrogen bonding. And the aggregated form generates a competitive photophysical process via ESIPT, causing the low *Φ*_PL_ value of PI-3 film [31]. When introducing only one hydroxyl group into the PMDA-based dianhydride moiety, the resulting PI film (PI-4, Figure 3a) displayed a blue-shifted emission band at 590 nm with a bigger large *Φ*_PL_ value of 0.068 and a bigger Stokes shift of 10,448 cm^−1^ compared with the PI-3 film [35]. 

**Figure 4:**
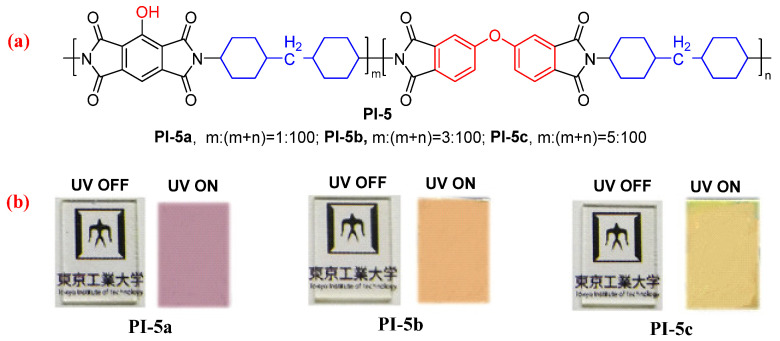
(**a**) The chemical structures of PI-5. (**b**) The photos of PI-5 films under sunlight and 365 nm UV light. Adapted with permission from Ref. [36]. Copyright 2021, ACS Publications.

As stated above, the formation of aggregates of hydroxyl-containing PMDA-based dianhydride moiety is unfavorable for improving the fluorescence quantum efficiency of PIs. In order to solve this problem of an aggregate formation of hydroxyl-containing PMDA-based dianhydride moiety, Ando et al. [36] continually synthesized a copolyimide (PI-5, Figure 4) using the hydroxyl-containing PMDA and ODPA dianhydrides, in which the hydroxyl-containing PMDA molar ratio was controlled at less than 5%. The PI-5 films demonstrated much higher *Φ*_PL_ value (0.20 for PI-5a and PI-5b, 0.23 for PI-5c) than that of the homo-PI film (0.09 for PI-1d, 0.068 for PI-4 [35]), which is due to the efficient energy transfer for ODPA and the suppressed aggregation by copolymerization.

**Figure 5:**
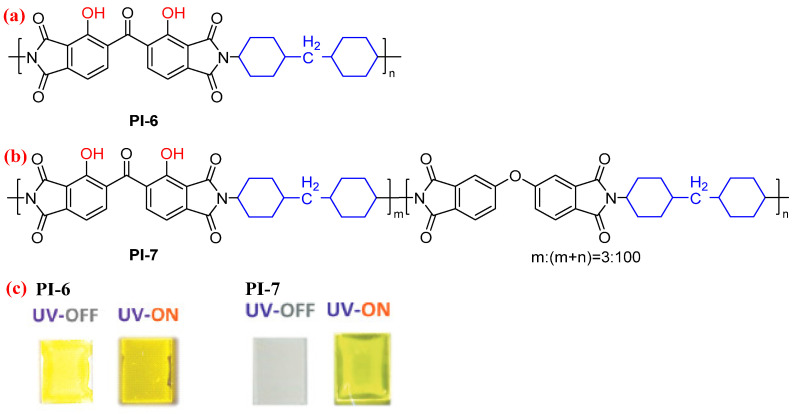
The chemical structures of (**a**) PI-6 and (**b**) PI-7. (**c**) Photos of PI-6 and PI-7 films under sunlight and UV light. Adapted with permission from Ref. [31]. Copyright 2021, Royal Society of Chemistry.

Similarly, Ando et al. [31] designed an another fluorescent semiaromatic PI containing two hydroxyl groups in BTDA-based dianhydride moiety (PI-6, Figure 5a). Compared with hydroxyl-containing PMDA-based PI (PI-3), the structure of PI-6 is more flexible owing to the rotatable –CO– linkage, which may effectively suppress aggregation of the dianhydride moieties. As a result, the *Φ*_PL_ value of PI-6 film (0.04) is higher than that of the PI-3 film (0.01). To achieve highly fluorescent PI film with high quantum yields, the authors continually designed a copolyimide (PI-7, Figure 5b) using the hydroxyl-containing BTDA and ODPA dianhydrides, whereby the molar ratio of the hydroxyl-containing BTDA dianhydride was set to 0.03. Although the molar fraction of the hydroxyl-containing BTDA dianhydride was considerably lower than that of ODPA, the PI-7 film exhibited prominent yellow fluorescence via ESIPT from the hydroxyl-containing dianhydride moiety (Figure 5c). The PI-7 film displays a sufficiently high quantum yield (*Φ*_PL_ = 0.14) due to the suppression of aggregation of the hydroxyl-containing BTDA dianhydride moieties.

## 3. Fluorescent PIs Containing Aromatic Diamine Segments

Often, fluorophores are introduced in the polymer chain to endow the fluorescence characteristics of polymer materials. However, the traditional aromatic PIs containing fluorophores show weak or no fluorescent characteristic due to the fluorescence quenching caused by reabsorption through the strong inter- and intramolecular CT effect in the aromatic PI chain [37]. Suppressing the CT effect in aromatic PIs is an effective method to improve the fluorescence quantum efficiency of aromatic PIs. Based on this, more and more fluorescent aromatic PIs created by appropriately controlling the electron push–pull effect between the aromatic diamine moieties and the dianhydride moieties have been reported.

### 3.1. PI Containing Mult-Phenylethylene Structure

As an efficient AIE functional unit, multi-phenylethylene (MPE, including Triphenylethylene, TriPE and Tetraphenylethylene, TetraPE) was introduced into the fluorescent small organic molecule or polymer to give them the AIE properties [38]. In addition, the aromatic diamine monomer containing the MPE structure possesses a nonplanar twisted and large conjugated structure. Therefore, introducing the MPE-based diamine monomers into the PI main chain can inhibit the intra- or intermolecular CT effects, effectively avoiding the fluorescence quenching of MPE-based PI caused by reabsorption through the CT effect. This an effective way to construct high-performance functional aromatic PIs with fluorescent characteristics.

**Figure 6:**
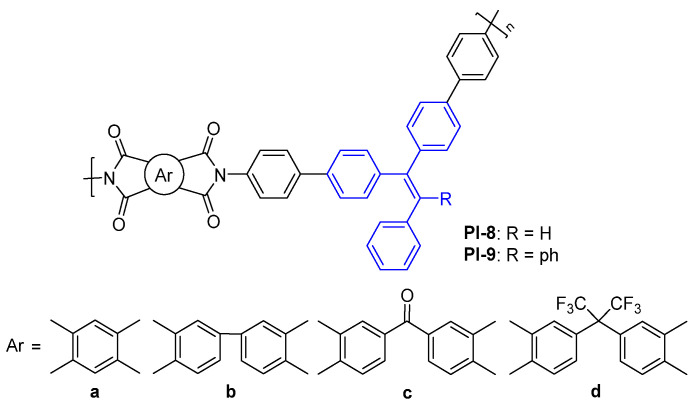
The chemical structures of PI-8-9.

For example, Zhang et al. designed and synthesized a series of high-performance functional aromatic PIs containing the TriPE (PI-8, Figure 6) [39] and TetraPE (PI-9, Figure 6) moieties [40] in diamine fragments. Thanks to the rigid nonplanar conjugated structure of the MPE structure, the intra- or/and intermolecular CT effects of PI-8 and PI-9 were effectively inhibited, resulting in the as-obtained PI showing fluorescence characteristics in the NMP solution and film state. Combining with other outstanding properties, such as low dielectric constant (*k*), light color, high glass transition temperatures (*T*_g_, more than 350 °C), high thermal stability, and excellent mechanical properties, these functional PIs show attractive potential applications in the field of high-performance flexible polymer photoconductive devices.

**Figure 7:**
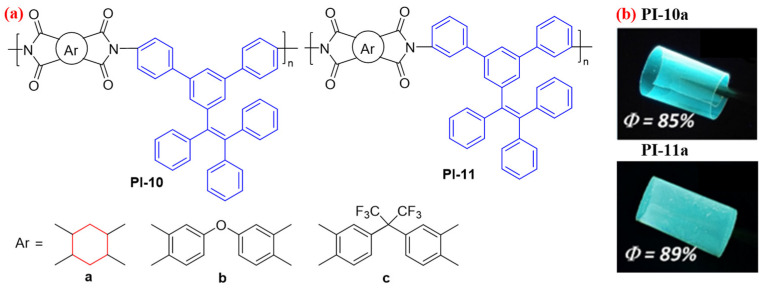
(**a**) The chemical structures of PI-10 (para-substituted) and PI-11 (meta-substituted). (**b**) Photos of PI-10a and PI-11a films under sunlight and 365 nm UV light. Adapted with permission from Ref. [41]. Copyright 2020, ACS Publications.

The fluorescence efficiency of the abovementioned aromatic PIs containing the MPE-based diamine fragment is relatively low due to the overall conjugation of the aromatic polymer chain. Therefore, Zhang et al. [41] continued to design two TetraPE-based diamine monomers, which were reacted with an aliphatic dianhydride 1,2,4,5-cyclohexanetetrcarboxylic dianhydride (HPMDA) to obtain two semiaromatic PIs with larger twisted side groups (PI-10a and PI-11a, Figure 7a). The intra- and intermolecular CT effects of the semiaromatic PIs were more effectively inhibited, and, thanks to the AIE performance of the TetraPE unit, the PI-10a and PI-11a films exhibited outstanding fluorescent characteristics with high absolute *Φ*_PL_ values of up to 89% for PI-11a (Figure 7b). However, the fully aromatic PI (PI-10b-c and PI-11b-c) films were of faint PL emission with absolute *Φ*_PL_ value lower than 4.8%. The authors demonstrated by theoretical calculation studies that these phenomena ascribed to the highly fluorescent AIE luminogen did not make contributions to the fluorescence intensity of fully aromatic PI films. Moreover, PI-10a and PI-11a also exhibit excellent thermal properties, such as high *T*_g_ (398 °C for PI-10a, 291 °C for PI-11a) and thermal decomposition temperature (*T*_d_) values (above 530 °C) and low CTE (below 50 μm·m^−1^·°C^−1^). The authors’ current experiment provided controllable ways for preparing high-thermal-stability polymeric materials with efficient fluorescence characteristics.

**Figure 8:**
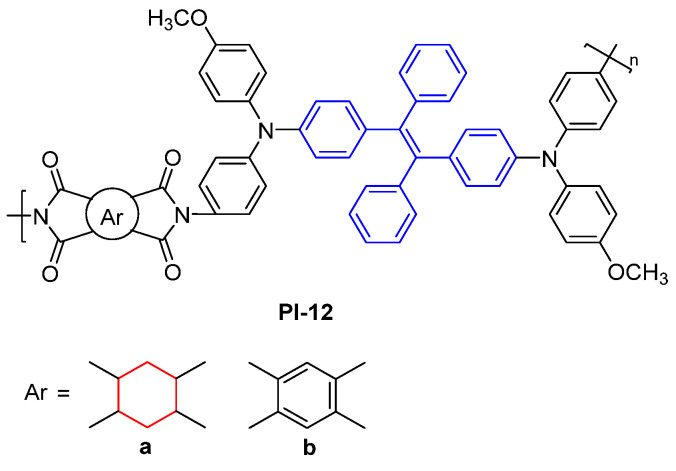
The chemical structures of PI-12.

Guan et al. [42] explored incorporating bulky or twisted TetraPE groups into the PI main chain to construct two fluorescent PIs (PI-12, Figure 8). Due to the effective inhibition of the inter- and intramolecular CT effects by bulky and twisted AIE groups, PI-12 exhibited fluorescence and AIE properties. The fluorescent intensity of PI-12a was significantly stronger than PI-12b, because the aliphatic structure reduced the CT effect on PI-12a. PI-12a and PI-12b exhibited weak PL emission at 530 and 418 nm with an absolute *Φ*_PL_ value of 1.4% and 0.4% in the NMP solution, respectively. Notably, due to the AIE activity, the PL emission of PI-12a and PI-12b films is greatly enhanced, with an absolute *Φ*_PL_ value of 10.4% and 1.4%, respectively.

**Figure 9:**
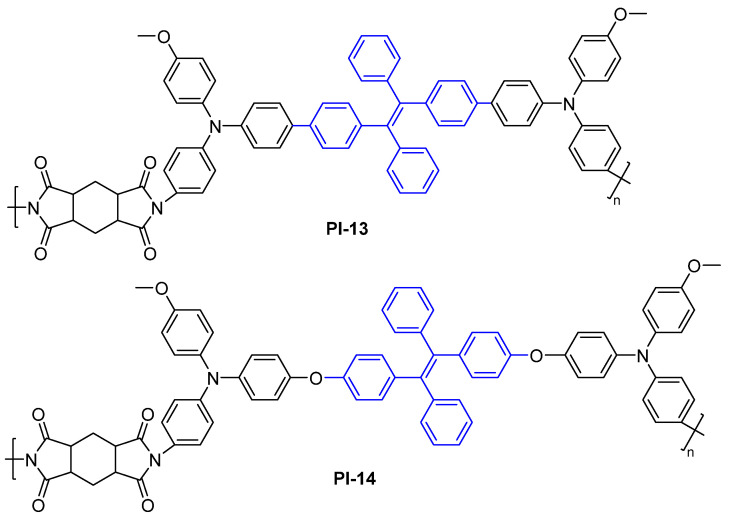
The chemical structures of PI-13 and PI-14.

Guan et al. [43] sequentially synthesized two fluorescent PIs containing different twisted TetraPE-based diamine and HPMDA fragments (PI-13 and PI-14, Figure 9). Similarly, PI-13 and PI-14 also exhibited fluorescence and AIE properties. However, the PI-13 film showed a lower *Φ*_PL_ value (1.4%) than that of the PI-12a film, due to PI-13 containing a longer conjugation. In PI-14, the ether linkage between the TetraPE and triphenylamine (TPA) unit completely interrupted the π-bridge conjugated structure. TPE existed as an independent fluorescent chromophore with a short conjugation structure in PI-14. Therefore, PI-14 exhibited a more localized π system, further blue-shifting the PL spectrum. The PI-14 film showed a higher *Φ*_PL_ value (2.6%) than that of the PI-13 film.

**Figure 10:**
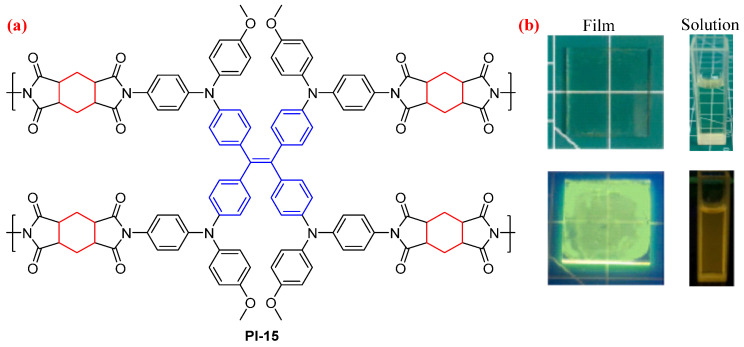
(**a**) The chemical structure of PI-15. (**b**) Photos of the PI-15 in THF solution and solid film under sunlight and 365 nm UV light. Adapted with permission from Ref. [44]. Copyright 2023, Elsevier.

Guan et al. [44] sequentially designed and prepared a hyperbranched fluorescent TetraPE-based PI (PI-15, Figure 10). The twisted TetraPE core and semiaromatic dianhydride could inhibit the CT effect. Therefore, the THF solution of PI-15 revealed a light-yellow fluorescence at 535 nm with a *Φ*_PL_ value of 2.2%. The PL emission of PI-15 was enhanced in the solid film with an absolute *Φ*_PL_ value of 4.7%, because PI-15 has the AIE property by introducing the TetraPE structure. 

All the abovementioned TetraPE-based PI films (PI-12-15) demonstrated excellent solubility and thermal stability. In addition, all the abovementioned TetraPE-based PIs possess a triphenylamine (TPA) unit. As an electrochromic/electrofluorochromic (EC/EFC) control unit, oxidation of the TPA unit can change the polymers from colorless to colored and quench the fluorescence from bright to dark state. Therefore, PI-12-15 showed good EC/EFC switching properties, which makes it a potential candidate in EC/EFC applications [42,43,44]. 

### 3.2. PI Containing Triarylamine Structure

Triarylamine (TAA) derivatives, such as Triphenylamine (TPA), possess a propeller-like structure and electron-donating characteristics, which make them a useful building block for constructing the AIE luminogen. However, directly introducing TPA-based diamine monomer into the PI main chain makes it difficult for TPA-based PI with the fluorescence characteristics to occur due to the high conjugated structure of the TPA-based PI chain [45]. Currently, researchers have found that introducing large conjugate side groups [46,47,48,49] or electron-withdrawing groups [50] in the noncoplanar fused TPA-based diamine monomer can effectively inhibit the CT effect in the TPA-based PI chain, thus allowing the construction of the fluorescent aromatic TPA-based PI.

**Figure 11:**
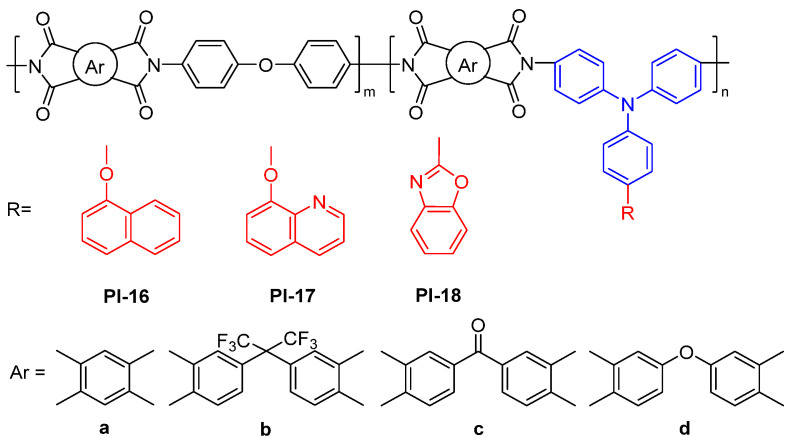
The chemical structures of PI-16-18.

For example, Siddiqi et al. [51] prepared a series of fluorescent copolyimides (PI-16, Figure 11) from a TPA-based diamine monomer containing a naphthoxy pendant group. The resulting PIs revealed the blue PL emission at 428–477 nm in the NMP solution with the *Φ*_PL_ values of 2.3–4.2% and 465–470 nm in the film state. The authors also prepared a series of fluorescent copolyimides from the TPA-based diamines containing 4-quinolin-8-yloxy (PI-17, Figure 11) [52] or benzoxazole (PI-18, Figure 11) [53] pendant groups. Similarly, these fluorescent copolyimides show lower PL efficiencies in the NMP solution, which could be attributed to the formation of the CT complex that may cause the nonradiative energy transfer, due to the high conjugated structure of the TPA-based monomer containing large conjugate side groups.

**Figure 12:**
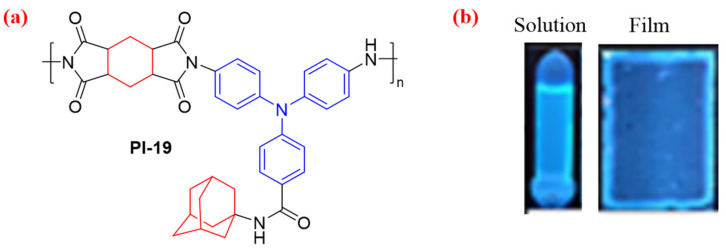
(**a**) The chemical structure of PI-19. (**b**) Photos of the PI-19 in film and solution under UV light. Adapted with permission from Ref. [54]. Copyright 2023, Elsevier.

Xu et al. [54] synthesized a PI (PI-19) by polycondensation of a TPA-based diamine monomer containing an amide bond bridged adamantane group (Figure 12). The cyclohexyl is a nonconjugated structure in TPA-based PI-19, which affects the CT transition and reduces the conjugation and CTC effect. Therefore, PI-19 has excellent transparency and PL emission. PI-19 solution and film show an emission peak around 442 nm with a *Φ*_PL_ value of 32.5% and 5.7%, respectively. In addition, the fluorescence intensity of PI-19 solution could be reduced in the presence of Fe^3+^, and this phenomenon is expected to be used as a specific Fe^3+^ probe.

**Figure 13:**
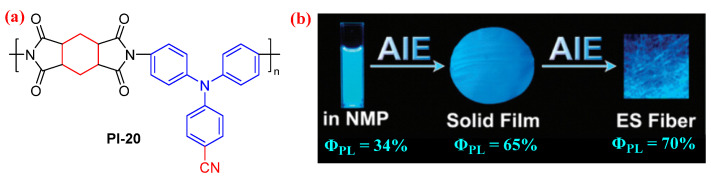
(**a**) The chemical structure of PI-20. (**b**) Photos of PI-20 in NMP solution, PI-20 solid film, and PI-20 ES fiber under 365 nm UV light. Adapted with permission from Ref. [55]. Copyright 2013, Royal Society of Chemistry.

Liou et al. [55] introduced an electron-withdrawing cyano group into the TPA-based diamine monomer (CN-TPA) and constructed a semiaromatic PI (PI-20, Figure 13a). The resulting PI-20 exhibited a blue PL emission at 443 nm with an absolute *Φ*_PL_ value of 34% in the NMP solution. Notably, the PL emission of PI-20 is greatly enhanced in the solid film with an absolute *Φ*_PL_ value of 65%, which could be further enhanced in the form of electrospun (ES) fiber with an absolute *Φ*_PL_ value of 70% (Figure 13b). These phenomena could be attributed to the AIE effect resulting from the introduction of the CN-TPA luminogen. These results demonstrate that the incorporation of the CN-TPA luminogen into high-performance polymers is a feasible approach to prepare efficient luminescent materials for optoelectronic applications.

**Figure 14:**
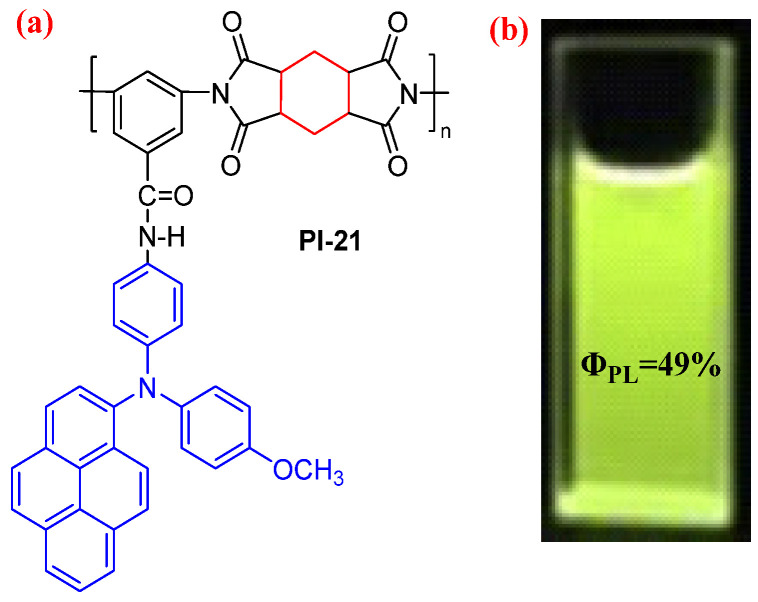
(**a**) The chemical structure of PI-21. (**b**) Photos of PI-21 in NMP solution (10 μM) under 365 nm UV light. Adapted with permission from Ref. [56]. Copyright 2019, Elsevier.

The ingenious introduction of the bulky pendant TAA-based fluorescent group into the PI side chain is also a useful way to construct the high-fluorescent PI. For example, Zhao et al. [56] prepared a fluorescent semiaromatic PI (PI-21, Figure 14a) by introducing the diphenylamine-pyrene unit into the PI side chain. The PI-21 presented a bright fluorescence emission at 541 nm with an absolute *Φ*_PL_ value of 49% in the NMP solution (Figure 14b). The high fluorescence efficiency of PI-21 is due to the bulky pendant diphenylamine-pyrene in the PI side chain loosening the packing and aliphatic dianhydride structure, reducing the CT effect.

The introduction of the dianhydride monomers containing the TAA unit into the PI backbone is also an effective way to construct the fluorescent PI, which suggest that the CT interactions would be efficiently decreased due to the electron-donating characteristics of the TAA unit.

**Figure 15:**
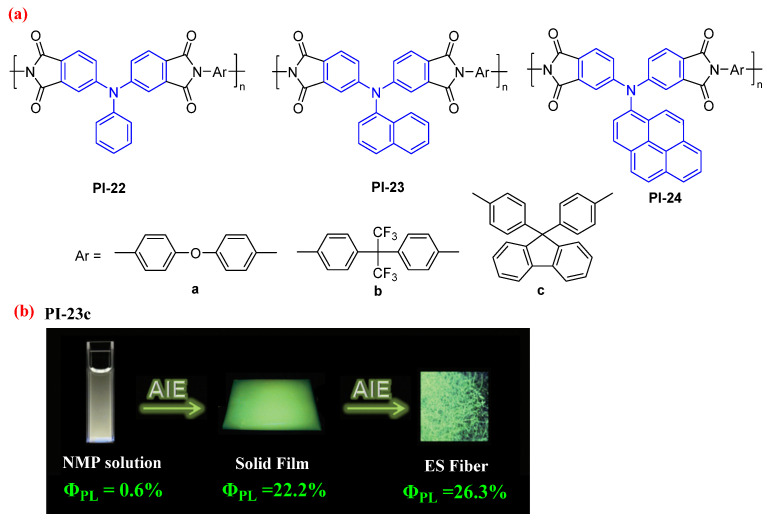
(**a**) The chemical structures of PI-22-24. (**b**) Photos of PI-23c in NMP solution, PI-23c solid film and PI-23c ES fiber under 365 nm UV light. Adapted with permission from Ref. [57]. Copyright 2013, Wiley.

For example, Liou et al. [57] prepared a series of fully aromatic PIs from the synthesized TAA-based dianhydrides (Figure 15a). As shown in Figure 15b, due to the AIE feature of TAA derivatives, these PIs exhibited weak yellowish-green PL emissions in NMP solutions with absolute *Φ*_PL_ values of 0.3–1.2% and high PL emissions in the solid state with absolute *Φ*_PL_ values up to 22% (PI-23c), which could be further enhanced in the form of ES fiber with the highest *Φ*_PL_ values of 26% (PI-23c). These results demonstrate that the incorporation of the TAA luminogen with an AIE feature as a dianhydride is an outstanding approaching to obtain the solid-state high-efficiency PL wholly aromatic functional PIs, which are desirable candidate materials for advanced optoelectronic applications.

**Figure 16:**
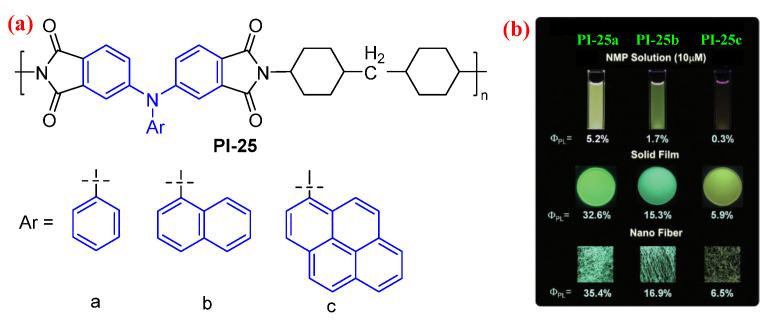
(**a**) The chemical structures of PI-25. (**b**) Photos of PI-25 in NMP solution, PI-25 solid films and PI-25 ES fibers under 365 nm UV light. Adapted with permission from Ref. [58]. Copyright 2015, Royal Society of Chemistry.

Subsequently, Liou et al. [58] replaced the aromatic diamine monomers with the aliphatic diamine monomer to prepared a series of the semiaromatic PI (PI-25) using the above-synthesized TAA-based dianhydride monomers. Compared to the fully aromatic TAA-based PI, the CT effect of the semiaromatic TAA-based PI is more suppressed, resulting in the higher fluorescence efficiency of the semiaromatic PI. The *Φ*_PL_ values of PI-25 were improved to 5.2%, 32.6%, and 35.4% in the NMP solution, solid film, and nanofiber states, compared to those of the fully aromatic TPA-based PIs of below 1.2%, 22.2%, and 26.3%, respectively (Figure 16b) [57]. 

**Figure 17:**
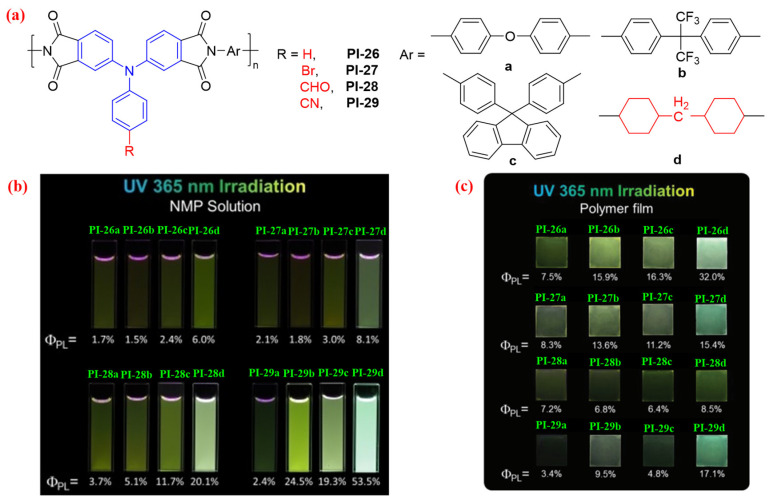
(**a**) The chemical structures of PI-26–29. (**b**) Photos of PI-26-29 in NMP solution (10 μM) under 365 nm UV light. (**c**) Photos of PI-26–29 solid films under 365 nm UV light. Adapted with permission from Ref. [59]. Copyright 2015, Royal Society of Chemistry.

Liou et al. [59] also prepared the fluorescent PIs (PI-26-29, Figure 17a) from TPA-based dianhydride monomers containing different substituted groups (-H, -Br, -CHO, and -CN) with various diamine monomers. The authors also found that the fully aromatic PIs, which have a relatively extensive CT effect, displayed lower *Φ*_PL_ than did the semiaromatic PIs (Figure 17b,c). In addition, PI-28 and PI-29 containing the strong electron acceptor (-CHO and -CN) showed higher *Φ*_PL_ in the NMP solution than did PI-26 and PI-27 (Figure 17b); however, in the film state, the *Φ*_PL_ values of PI-28 and PI-29 were observed to be similar to those of PI-26 and PI-27 (Figure 17c). The hybridized local and charge-transfer transitions could be enhanced by introducing the strong electron acceptor into the TPA luminogens to exhibit higher *Φ*_PL_ in the solution state, but also by facilitating intermolecular CT and energy transfer interactions in the solid film state, which leads to the ACQ effect and results in lower *Φ*_PL_. These fluorescent PIs have great potential for high-performance optoelectronic applications.

**Figure 18:**
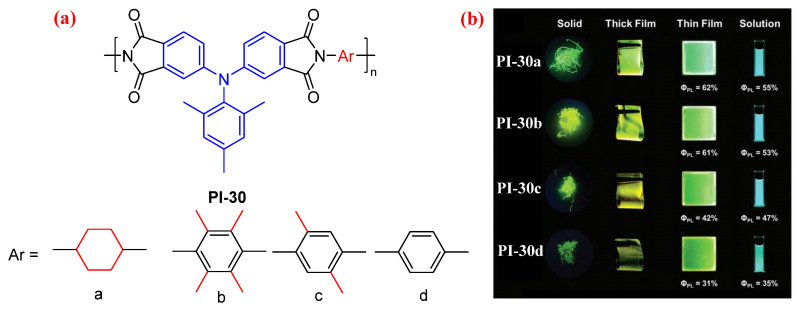
(**a**) The chemical structures of PI-30. (**b**) Photos of PI-30 in solid, thick film (thickness: ≈35 µm), thin film (thickness: ≈90 nm), and solution state under 365 nm UV light. Adapted with permission from Ref. [60]. Copyright 2022, Wiley.

The fluorescence efficiency of semiaromatic PIs derived from TPA-based dianhydride is higher than that of the aromatic PI from the same dianhydride. However, the aliphatic diamine structure in these TPA-based PIs reduced *T*_g_. In order to develop fluorescent aromatic PIs with high fluorescence efficiency and high *T*_g_, Liou et al. [60] prepared three aromatic PIs derived from TPA-based dianhydride containing three methyl groups and methyl-substituent *p*-phenylenediamine (PI-30b–d, Figure 18a). Introducing methyl groups into TPA-based dianhydride and *p*-phenylenediamine to form a bulky and highly twisted conformation efficiently hindered π–π stacking and reduced the intermolecular CT effect of the resulting aromatic PIs. The fluorescence efficiency of these aromatic PIs was increased with an increased number of methyl groups in the diamine (Figure 18b). Among these aromatic PIs, the PI-30b exhibited the highest *Φ*_PL_, up to 53% in the solution state and 61% in the film state, which was similar to that of the semiaromatic PI-30a derived from the same dianhydride and alicyclic diamine (55% in the solution state and 61% in the film state). The authors speculated that the tetramethyl-substituted *p*-phenylenediamine can effectively suppress intramolecular CT by playing the role of the aliphatic unit. In addition, these aromatic PIs exhibited extremely high *T*_g_ (above 400 °C); in particular, PI-30b exhibited the highest *T*_g_, to 470 °C.

### 3.3. PI Containing Triarylmethane Structure

Triarylmethane-based (TAM-based) diamine monomers containing an sp^3^ hybridized C atom separate the conjugated pendant aryl group from the PI main chain, which dispels the influence of the intramolecular CT effect of PI on the pendant aryl group. Therefore, introducing the TAM-based diamine monomer into the PI main chain is an effective strategy to construct the fluorescent PIs [61,62]. 

**Figure 19:**
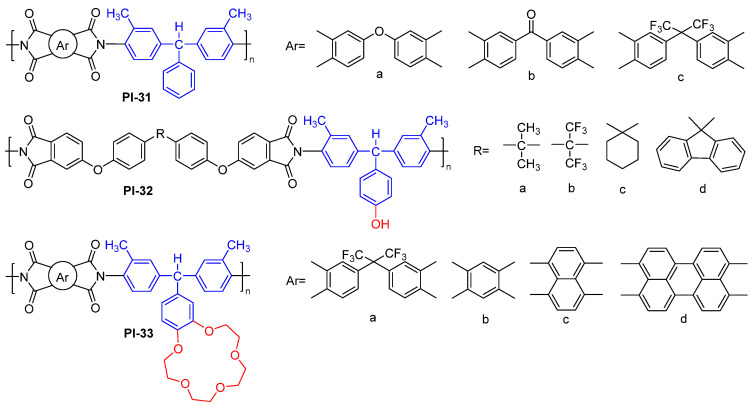
The chemical structures of PI-31, PI-32, and PI-33.

For example, Damaceanu et al. synthesized a series of fluorescent triphenylmethane-based (TPM-based) PIs with the pendant phenyl [63] (PI-31) or hydroxyl-functionalized phenyl [64] (PI-32) in the TPM core (Figure 19). However, due to the low conjugation of a single pendant phenyl, these TPM-based PI systems exhibited weak fluorescence in the solution phase and no fluorescence in the film state. Then, the authors synthesized the TPM-based PI functionalized group with benzo-15-crown-5 ether in the pendant phenyl of the TPM core (PI-33, Figure 19) [65]. Due to a ground-state intermolecular CT complex between the donor-crown-ether modified TPM diamine and the acceptor diimide segment, these PIs displayed strong light emission in the green to yellow spectral range in the THF solution. Moreover, since crown ethers can be complexed with metal ions, these PIs can serve as fluorescent sensors for the detection of cations like Li^+^, Na^+^, and K^+^.

**Figure 20:**
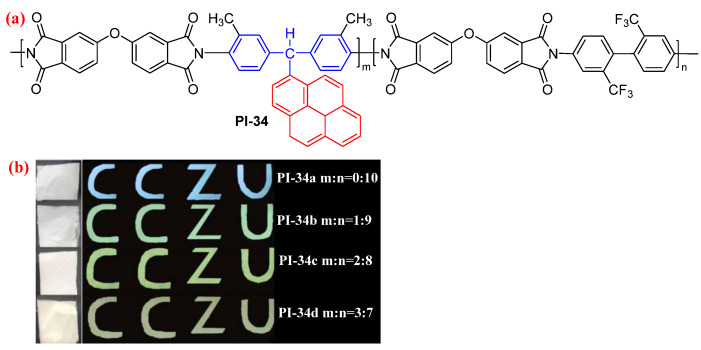
(**a**) The chemical structure of PI-34. (**b**) Photos of PI-34 film under sunlight and 365 nm UV light. Adapted with permission from Ref. [66]. Copyright 2020, Elsevier.

Zhao et al. [66] designed a TAM-based diamine monomer containing the high conjugated pyrene group, which was reacted with 2,2′-bis(trifluoromethyl) benzidine and ODPA in different mole ratios for obtaining the copolyimides (PI-34, Figure 20a). In the DMAc solution, the fluorescence intensity of PI-34 was gradually enhanced with increasing pyrene group content, because the rigid nonplanar conjugate structure made the polymer have a large steric hindrance effect, inhibiting the CT effect between the polymer molecules. In addition, the electrospun fiber membranes still possessed strong fluorescence intensity (Figure 20b). The as-obtained aromatic PI can provide a novel cost-effective functional material and a simple preparation process for flexible luminescent devices.

**Figure 21:**
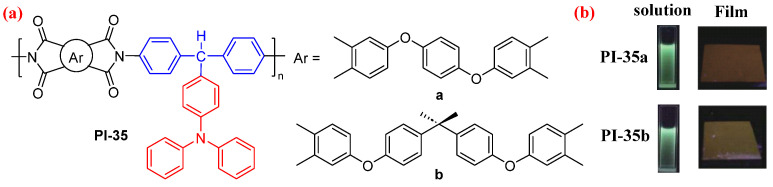
(**a**) The chemical structures of PI-35. (**b**) Photos of PI-35 in NMP solution and films under UV light. Adapted with permission from Ref. [45]. Copyright 2018, Acta Polymerica Sinica.

Similarly, Zhang et al. [45] designed a TAM-based diamine monomer containing a pendant TPA group, which was polymerized with two dianhydrides to form two fluorescent TAM-based PIs (PI-35). The sp^3^ hybridized carbon atom in the TAM-based system separates the pendant TPA group from the PI main chain, which dispels the influence of the intramolecular CT effect of the PI on TPA. Furthermore, the relatively independent TPA group can have a strong intermolecular CT interaction with the PI main chains, which leads this system to exhibit a strong fluorescence. Therefore, by increasing the concentration of the as-obtained aromatic PIs in the NMP solution, their emission changes from nonluminescence to bright blue emission, followed by a redshift due to the formation of the strong intermolecular CT effect. PI-35a and PI-35b show green photoluminescence at 504 nm and 508 nm in the NMP solution and bright orange photoluminescence at 565 and 585 nm in their films, respectively (Figure 21b).

### 3.4. PI Containing Fluorene-Based Cardo Structure

The fluorene moiety is an efficient fluorescent chromophore, and fluorene-based cardo diamine monomers possess an sp^3^ hybridized C atom. Therefore, introducing a fluorene-based cardo diamine monomer into the PI main chain would cut off the conjugated effect of the rigid planar fluorene side chain from the polymer main chain, and, thus, minimize the intramolecular CT effect to construct fluorescent PI.

**Figure 22:**
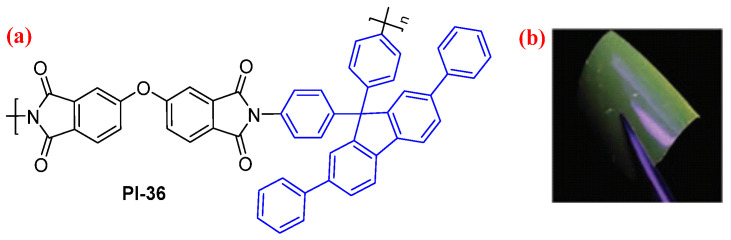
(**a**) The chemical structure of PI-36. (**b**) Photo of PI-36 film under UV light. Adapted with permission from Ref. [67]. Copyright 2017, Royal Society of Chemistry.

For example, Zhang et al. [67] synthesized a diamine containing a pendant tetraphenyl fluorene moiety (TPF), and the corresponding aromatic PIs (PI-36, Figure 22a) were prepared. PI-36 with a fluorene unit in the side chain emits a considerably high intensity blue–green emission at 492 nm in the film state (Figure 22b). In addition, the configurations of sandwich memory devices with the PI-36 as the active layer were fabricated successfully, and showed volatile static random access memory type switching behavior.

**Figure 23:**
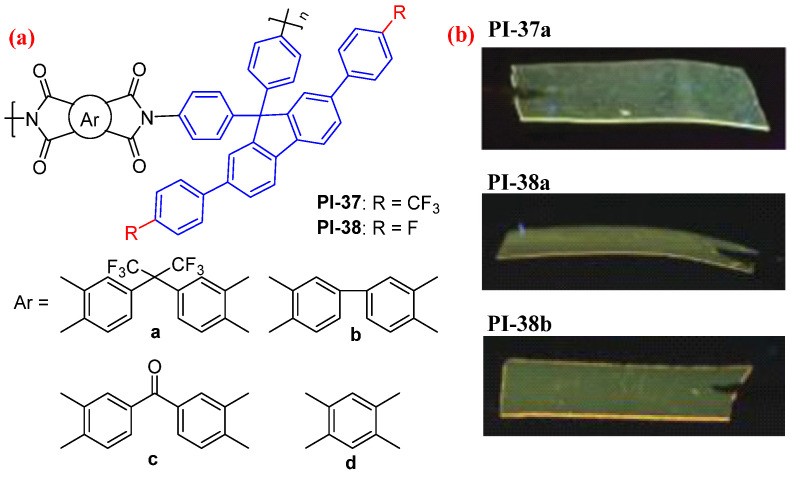
(**a**) The chemical structures of PI-37 and PI-38. (**b**) Photos of PI-37-38 films under 365 nm UV light. Adapted with permission from Refs. [68,69]. Copyright 2017, Royal Society of Chemistry and 2019, Springer Link, respectively.

Zhang et al. further introduced the low-polarizability groups (CF_3_ group [68] or F atom [69]) into the TPF-based diamine and synthesized a series of high-performance multifunctional PIs (PI-37 and PI-38, Figure 23a). PI-37 exhibited obvious fluorescence characteristics at 482–503 nm in the NMP solution and 546–560 nm in the film state. For PI-38, the maximum fluorescence emission was observed at 422–424 nm in the NMP solution and at 470–548 nm in the film state. Furthermore, these PI films showed exceptional thermostability (*T*_g_ of PI-37d as high as 494 °C), solubility, light color (some even colorlessness), high transparency, and ultralow dielectric constant (≈1.93 for PI-37a), due to introducing the CF_3_ groups or F into the rigid nonplanar conjugated fluorene moiety.

**Figure 24:**
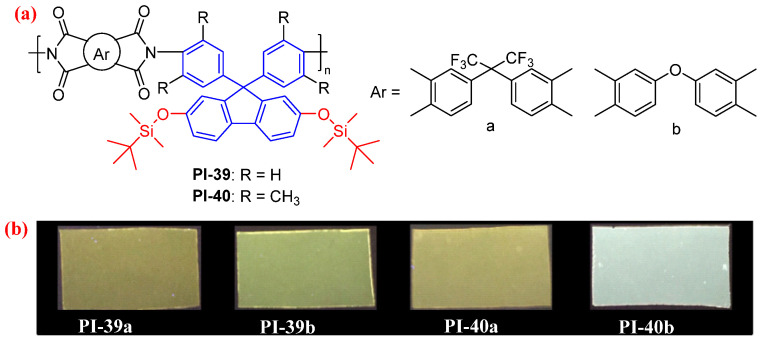
(**a**) The chemical structures of PI-39 and PI-40. (**b**) Photos of PI-39 and PI-40 films under 365 nm UV light. Adapted with permission from Ref. [70]. Copyright 2020, Elsevier.

Our group synthesized a series of fluorene-based PIs with *tert*-butyldimethylsiloxy (TBS) side groups (PI-39 and PI-40, Figure 24a) by a direct silyl ether reaction of fluorene-based cardo-PI-containing OH groups [70]. When the electron-donating -OH groups in the PI chains are replaced with bulky TBS groups, the intermolecular attraction forces (hydrogen bonding) and the molecular chain stacking are reduced; thus, the interchain CT effect of the PI-39 and PI-40 can be partly depressed. In contrast with hydroxyl-containing fluorene-based cardo PI films with no fluorescent characteristic, these PI-39 and PI-40 films exhibit bright fluorescence at 466–526 nm with *Φ*_PL_ values of 3.5–4.8%. In addition, these PI films still possess excellent thermal properties with a *T*_g_ of 311.2–338.9 °C, a light color with high optical transparence (over 80% at 450 nm), and a low dielectric constant (as low as 2.44 at 1 MHz for PI-40a). This work provides a convenient way to prepare multifunctional PI films with excellent overall properties, which shows the high potential applications of these PI films in microelectronic, electric, and photonics industries.

**Figure 25:**
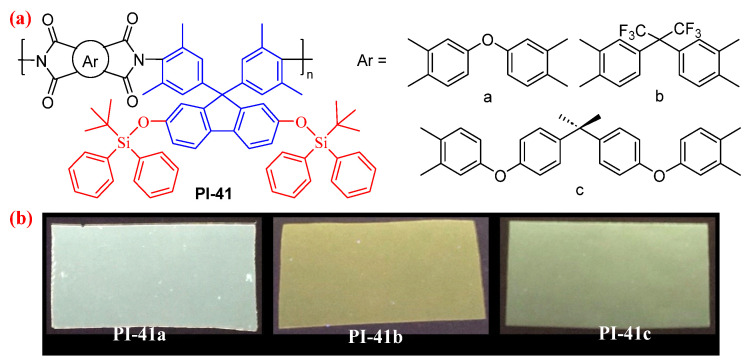
(**a**) The chemical structures of PI-41. (**b**) Photos of PI-41 films under 365 nm UV light. Adapted with permission from Ref. [71]. Copyright 2022, ACS Publications.

When introducing the *tert*-butyldiphenylsilyl (TBDPS) side groups into fluorene-based cardo PI (PI-41, Figure 25), the *Φ*_PL_ values of PI-41 films further increased to 5.2–9.3% compared with those of PI-40 films [71]. In addition, these fluorescent *tert*-butyldimethylsiloxy-containing fluorene-based PI films can be used to detect F^-^ based on the sensing mechanism of the cleavage of the Si–O bond [72]. 

**Figure 26:**
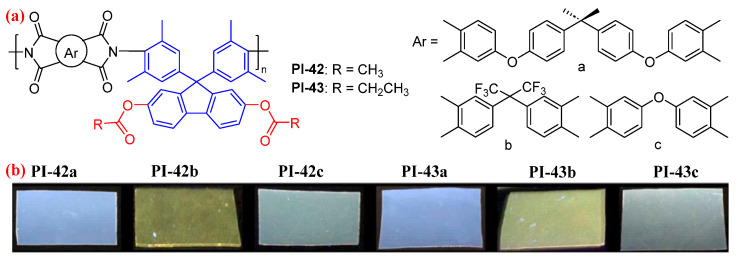
(**a**) The chemical structures of PI-42 and PI-43. (**b**) Photos of PI-42 and PI-43 films under 365 nm UV light. Adapted with permission from Ref. [73]. Copyright 2021, Elsevier.

Similarly, our group then synthesized fluorene-based PIs (PI-42 and PI-43, Figure 26) with an acyloxy group by post-polymerization modification on hydroxyl-containing fluorene-based cardo PIs [73]. The incorporation of electron-withdrawing acyloxy groups may reduce the packing density of PI chains and weaken both intra- and intermolecular CT effects. Accordingly, the resulting PI-42 and PI-43 films exhibited a bright fluorescence at 436–493 nm with *Φ*_PL_ values of 3.8–7.5%. In addition, the incorporation of acyloxy groups bestows the resulting PI films with light color, high optical transmittance of 80.1–83.5% at 400 nm, and low *k* of 2.58–2.90 at 1 MHz, and no obvious reduction in the *T*_g_ of the resulting PI films (301.1–390.9 °C).

### 3.5. PI Containing Nitrogen Heterocyclic Fluorophore

Many nitrogen-containing heterocyclic rings are common fluorophores, which are often used to construct the fluorescent polymer. Therefore, the reasonable molecular design of the diamine monomer containing nitrogen heterocyclic fluorophore to appropriate control of the intramolecular CT effects between the diamine and dianhydride moieties is an effective strategy to construct the fluorescent PIs [23,74,75]. 

**Figure 27:**
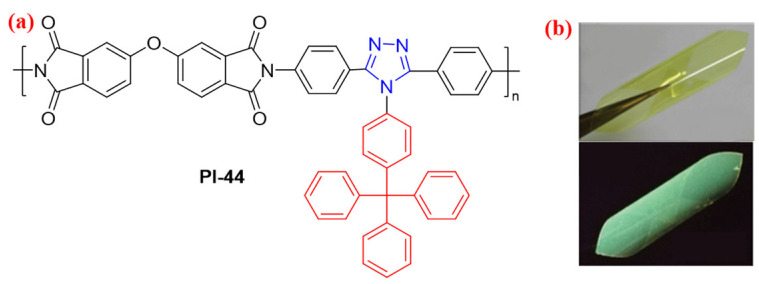
(**a**) The chemical structures of PI-44. (**b**) Photos of the flexible PI-44 film under sunlight and 365 nm UV light. Adapted with permission from Ref. [78]. Copyright 2016, Royal Society of Chemistry.

The triazole structure possesses the electron-withdrawing effect, which could be introduced into the diamine fragment to prepare the highly fluorescent aromatic PI, due to the weakened CT effect by the triazole unit [76,77]. For example, Zhang et al. [78] synthesized a triazole-based PI containing large nonplanar and nonpolar tetraphenylmethane side groups (PI-44, Figure 27a). Because the electron acceptor triazole groups may help to weaken the intramolecular CT interaction, the resulting PI showed bright green fluorescence with a high *Φ*_PL_ value of 61% in a dilute solution of CH_2_Cl_2_ and 13% in the film state (Figure 27b). This as-obtained aromatic PI with high fluorescence may be the next generation of high-performance flexible light-emitting materials.

**Figure 28:**
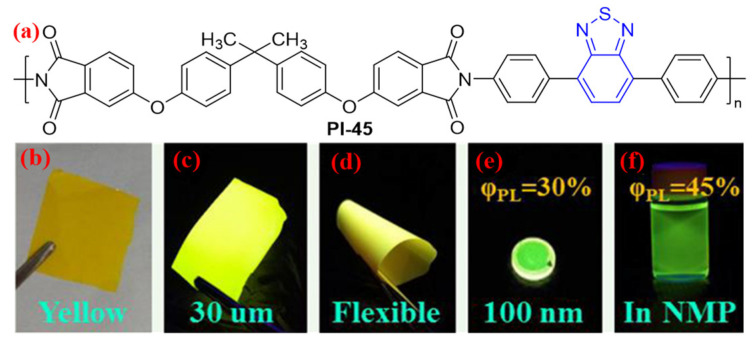
(**a**) The chemical structure of PI-45. (**b**) PI-45 film under sunlight; (**c**,**d**) flexible PI-45 film (30 μm) under 365 nm UV light; (**e**) PI-45 thin film coated on the quartz plate (100 nm) under 365 nm UV light; (**f**) PI-45 in NMP solution (2 × 10^−2^ mg·mL^−1^) under 365 nm UV light. Adapted with permission from Ref. [79]. Copyright 2018, ACS Publications.

Similarly, Zhang et al. [79] synthesized a benzothiadiazole-based diamine with a strong electron-withdrawing effect, which was polymerized with BPADA to form a fluorescent aromatic PI (PI-45, Figure 28a). Due to the strong electron-withdrawing effect of the benzothiadiazole moiety in the diamine fragment, it tends to suppress intramolecular CT effects, endowing the resulting PI with high fluorescence efficiency. The *Φ*_PL_ value of PI-45 is 45% in the NMP solution and 30% in the film state (Figure 28b–f). In addition, ITO/PI-45/Au shows nonvolatile “write once-read many” characteristics in a memory device.

**Figure 29:**
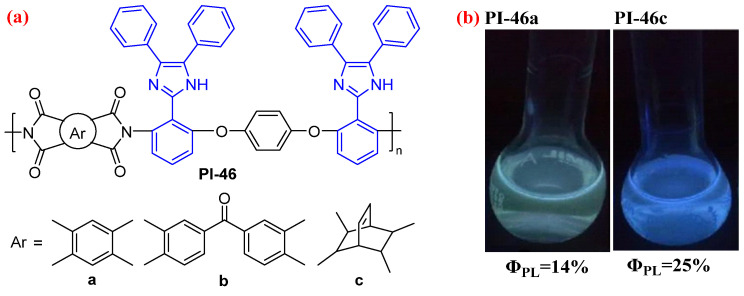
(**a**) The chemical structures of PI-46. (**b**) Photos of the flexible PI-46a and PI-46c solutions under sunlight and 365 nm UV light. Adapted with permission from Ref. [82]. Copyright 2010, Springer Nature.

It is well known that the arylimidazole structure has fluorescence properties. Introducing the bulky arylimidazole pendant group into the PI chain can prevent fluorescence quenching [80,81]. For example, Ghaemy et al. [82] synthesized a triaryl imidazole-based diamine, which was reacted with aromatic and cycloaliphatic dianhydrides to obtain three fluorescent PIs (PI-46, Figure 29). PI-46 has a bulky diaryl imidazole substituent in diamine moiety, which limits close packing between aromatic chromophores in the solid state. This can effectively minimize the undesirable intramolecular CT and interchain interactions. Therefore, PI-46 shows emission at 431–464 in the solid state and at 423–459 nm in a dilute DMAc solution with *Φ*_PL_ values in the range of 11–25%.

**Figure 30:**
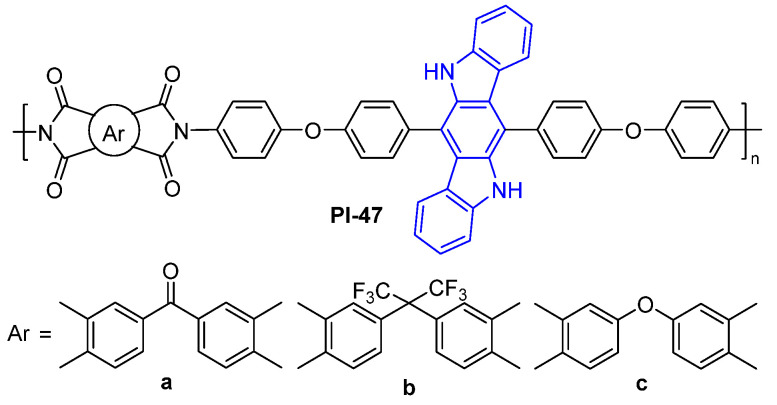
The chemical structures of PI-47.

Introducing a twisted noncoplanar indolocarbazole structure into the diamine moiety is also an effective strategy to prepare the fluorescent PI. For example, Li et al. [83] synthesized a diamine monomer containing a twisted noncoplanar indolocarbazole, and the corresponding aromatic PIs were prepared (PI-47, Figure 30). The authors found that PI-47 showed blue light emission at around 448 nm in the NMP solution. In the film state, the fluorescence of PI-47 still maintains certain fluorescence performance with a maximum emission wavelength at about 451 nm, but fluorescence is quenched dramatically.

**Figure 31:**
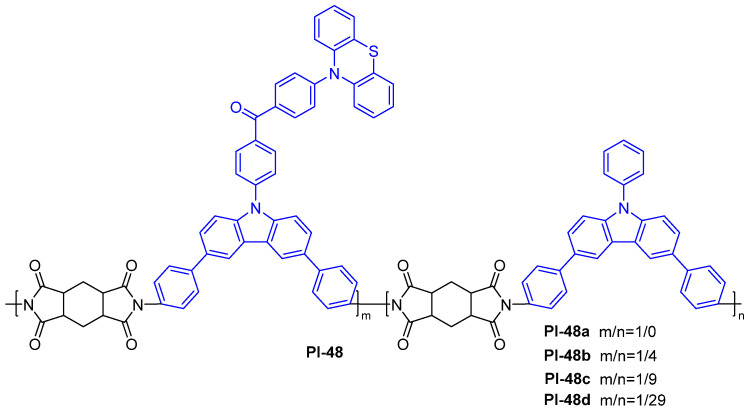
The chemical structure of PI-48.

Zhang et al. [23] reported a series of nonconjugated rigid PI-based fluorescent polymers with a thermally activated delayed fluorescence (TADF) property (PI-48, Figure 31), which were implemented by using a new diamine containing an asymmetrical carbazole and phenothiazine heterocyclic TADF luminous core structure as the TADF unity, carbazole-containing diamine as the host moiety, and dianhydride HPMDA as the linker unit. The nonconjugated aliphatic ring can not only improve its solubility, but it also effectively inhibits the CT effect and makes the electron cloud localized on the TADF unit, thus enabling the polymer to inherit the TADF property. The PI-48 films doped with 50 wt.% mCP show higher-efficiency fluorescence, with *Φ*_PL_ values of 57.1–86.7%. Importantly, highly efficient solution-processed OLEDs were achieved by adopting PI-48, resulting in external quantum efficiency higher than 5% (up to 21.0% for PI-48b).

**Figure 32:**
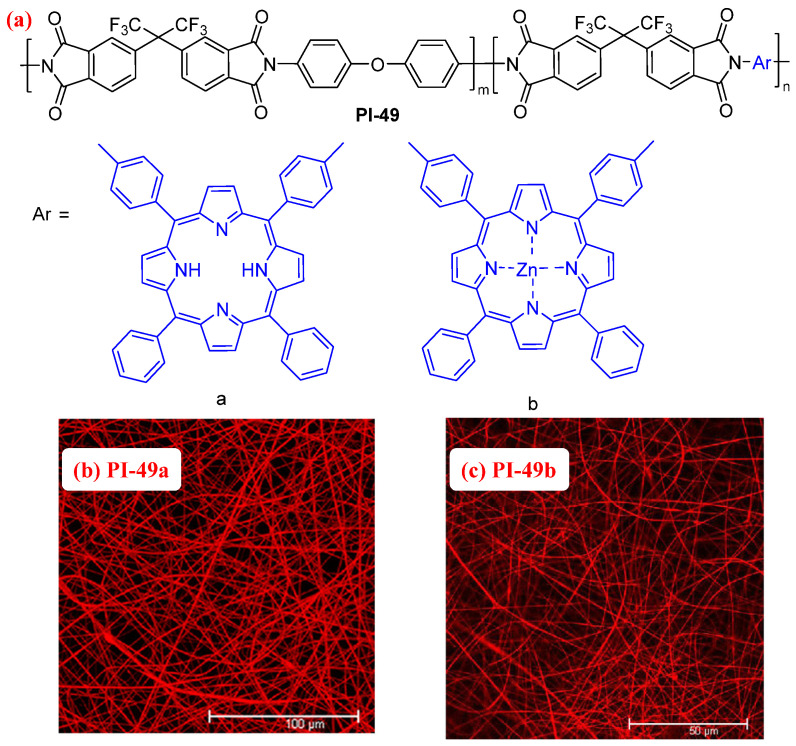
(**a**) The chemical structures of PI-49, and confocal laser scanning microscopy images of (**b**) PI-49a and (**c**) PI-49b nanofibrous membranes. Adapted with permission from Refs. [84,87]. Copyright 2010 and 2013, Elsevier.

Porphyrin is a common fluorophore. However, it is well known that the aggregation of porphyrin chromophores causes fluorescence quenching, which detrimentally affects the application of fluorescent materials [84]. Introducing porphyrin fluorophores into the PI main chains reduces the aggregation-caused fluorescence self-quenching of porphyrin [85,86]. For example, Wu et al. [84] introduced the porphyrin moieties into the PI backbones, which can be used to construct the fluorescent copolyimide (PI-49a, Figure 32a). The PI-49a nanofibrous membrane prepared by electrospinning emitted a uniform red light (Figure 32b). Due to the protonation of the neutral porphyrin moieties in PI-49a, the PI-49a can be used as the fluorescent sensor for the detection of HCl gas. Similarly, the authors then prepared a porphyrinated PI complexed with Zn (PI-49b, Figure 32a), which can also maintain the red fluorescence in the nanofibrous membrane (Figure 32c) and can be used to detect TNT [87]. 

**Figure 33:**
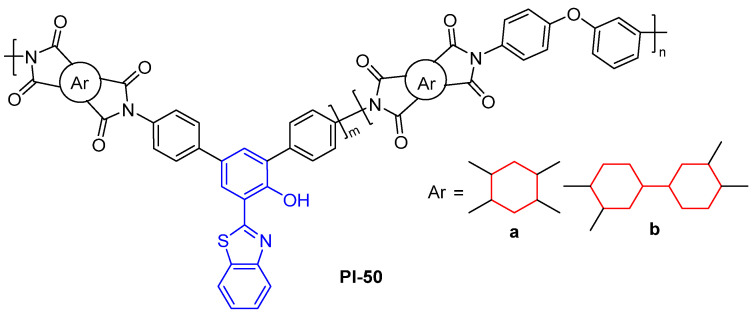
The chemical structures of PI-50.

As an ESIPT fluorophore, 2-(2′-hydroxyphenyl)benzothiazole (HBT) unit is able to be introduced into PI chains to construct the fluorescent PI. For example, Zhang et al. [88] designed and synthesized a series of fluorescent copolyimides (PI-50, Figure 33) from the HBT-based diamide. The as-prepared PI-50 films retained the ESIPT performance with a large Stokes shift of larger than 8105 cm^−1^, a high photoluminescence quantum efficiency of up to 29%, decent colorless and transparency, high thermal performance (*T*_d5%_ = 468 °C and *T*_g_ ˃ 260 °C), and solubility in both conventional aprotic and protic solvents. The developed PIs provided good examples for the development of ESIPT polymers with controllable emission wavelengths as well as high thermal, environmental, and radiation stability.

### 3.6. PI Containing Oxygen-Containing Heteroatom Fluorophore

Similarly, the reasonable molecular design of the diamine monomer containing oxygen-containing heterocyclic fluorophore to appropriate control of the intramolecular CT effects between the diamine and dianhydride moieties is also an effective strategy to construct fluorescent PI.

**Figure 34:**
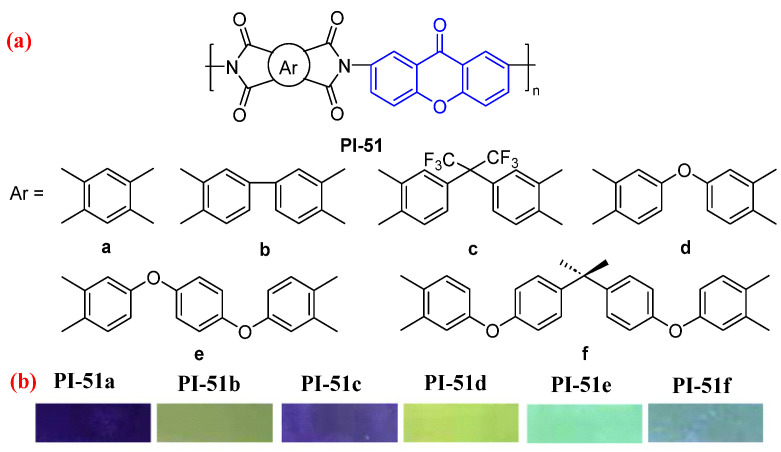
(**a**) The chemical structures of PI-51. (**b**) Photos of PI-51 films under 365 nm UV light. Adapted with permission from Ref. [89]. Copyright 2022, Elsevier.

As an oxygen-containing fluorophore group, xanthone moiety is introduced into PI main chains to provide the fluorescence performance. For example, Qiu et al. [89]. prepared a series of aromatic PIs containing xanthone fluorophore (PI-51, Figure 34a). PI-51 films exhibited distinct fluorescence emission at 443–552 nm with absolute *Φ*_PL_ values of 0.3–1.9%, except for PI-51a, which hardly emitted fluorescence. Compared with those of PI-51a–c, the fluorescence intensity and absolute *Φ*_PL_ of PI-51d–f improved significantly. This may be explained by the strong intramolecular CT effect between the diamine and the strong electron-absorbing dianhydride in PI-51a–c quenching or attenuating the fluorescence, while the weak electron-absorbing dianhydride in PI-51d–f inhibited the intramolecular CT effect, leading to a strong fluorescence emission. Among these PI films, PI-51d exhibited the highest fluorescence intensity and absolute *Φ*_PL_ values of 1.9%. 

**Figure 35:**
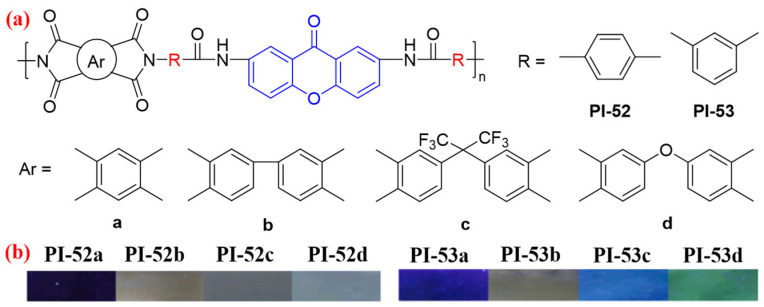
(**a**) The chemical structures of PI-52 and PI-53. (**b**) Photos of PI-52 and PI-53 films under 365 nm UV light. Adapted with permission from Ref. [90]. Copyright 2022, Elsevier.

Next, Qiu et al. [90] continued to design two diamines containing amide and rigid xanthone groups, which were reacted with four conventional dianhydride monomers to obtain two series of fluorescent PI films (PI-52 and PI-53, Figure 35). The authors found that the absolute *Φ*_PL_ values of PI-52 films (0.3–1.5%) is generally lower than that of PI-53 film (0.3–2.9%). This is because PI-52 films prepared by *para*-substituents diamine monomers have more hydrogen bonding, closer molecular chain packing, and stronger molecular CT effect. However, the PI-52 film’s *T*_g_, dimensional stability, and mechanical properties are better than PI-53 films. Therefore, the introduction of rigid xanthone groups and amide structures in the PI main chain can endow the PI with fluorescence performance and simultaneously improve the PI’s *T*_g_ and reduce CTE. Among them, PI-52d and PI-53d films prepared by ODPA have the strongest fluorescence and emit green fluorescence under UV excitation, and their absolute *Φ*_PL_ values are 1.5% and 2.9%, respectively.

**Figure 36:**
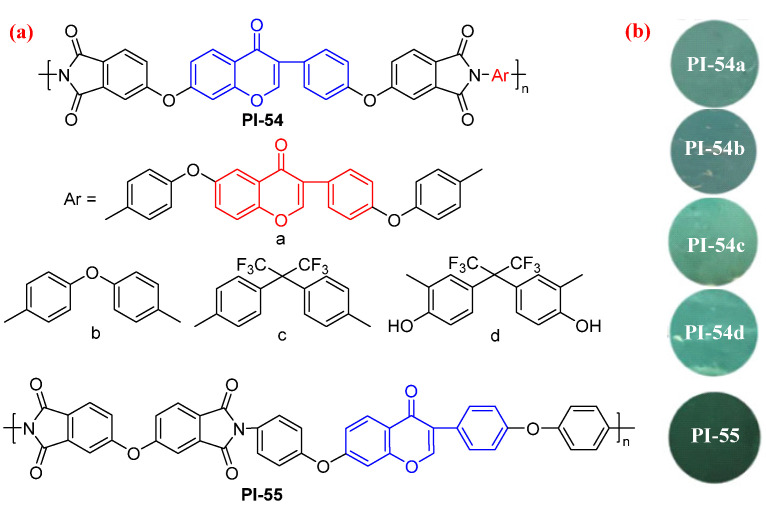
(**a**) The chemical structures of PI-54 and PI-55. (**b**) Photos of PI-54 and PI-55 films under 365 nm UV light. Adapted with permission from Ref. [91]. Copyright 2023, ACS Publications.

Yan et al. [91] synthesized a series of daidzein-based PIs (PI-54 and PI-55, Figure 36) with a high biobased content of up to 53%. The as-obtained daidzein-based PIs showed excellent fluorescence characteristics with absolute *Φ*_PL_ values of 6.3–14.1%. DFT calculations indicate an effective inter- and intramolecular conjugation and the inhibition effect of intramolecular charge transfer by “enone and ether bonds” in daidzein-derived PIs. Therefore, the daidzein-based monomers can promote the fluorescence effect of PIs. In addition, these PI films also possess superior mechanical strength and remarkable thermal stability, along with good optical properties. This material is not only an ideal candidate for the substitution of petroleum PIs, but it also has the potential for application as a photosensor.

### 3.7. PI Containing Sulfone Group

**Figure 37:**
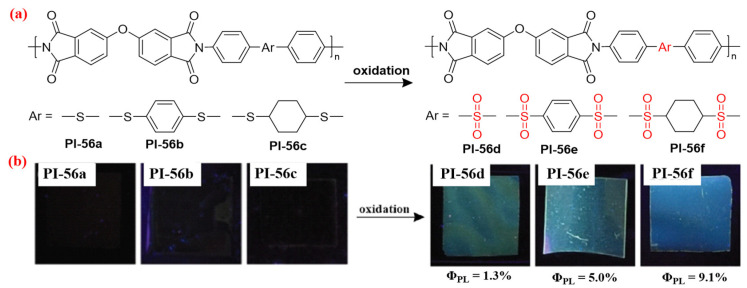
(**a**) The chemical structures of PI-56. (**b**) Photos of PI-56 films under 365 nm UV light. Adapted with permission from Ref. [92]. Copyright 2017, Royal Society of Chemistry.

As the electron acceptor, the sulfone group could be introduced into the diamine fragment to prepare the highly fluorescent aromatic PI, due to the weakened CT effect by the sulfone group. For example, Zhang et al. [92] firstly designed and synthesized three nonfluorescent sulfide-containing PI films. After being immersed in an H_2_O_2_ solution, these PI films could emit fluorescence under UV light irradiation, because of the PI molecular structure turning the sulfide (donor) into sulfone (acceptor) in the diamine moiety (Figure 37a). A boosted fluorescent *Φ*_PL_ value up to 9.1% (PI-56f) was found to be contributed by CT suppression along the main chain through oxidation-induced sulfide-to-sulfone transformation (Figure 37b).

## 4. Fluorescent Microporous PI Bond-Linked Networks or Covalent Organic Frameworks (COFs)

The 2D aromatic porous networks or COFs show strong fluorescence, due to their large π–π conjugate, inherent rigid structure [93]. Therefore, the large π–π conjugate structures of PI bond-linked porous networks or COFs are expected to be the new strong fluorescent materials.

**Figure 38:**
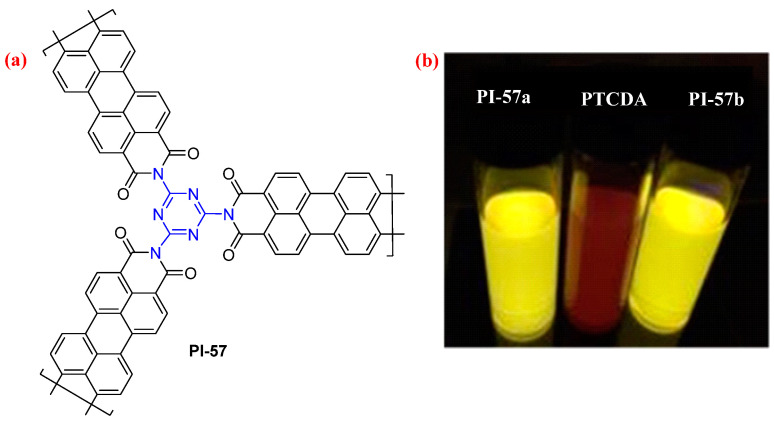
(**a**) The structure of PI-57. (**b**) Photos of PI-57 and PTCDA as THF dispersions (0.5 g·L^−1^) under 365 nm UV light. Adapted with permission from Ref. [94]. Copyright 2015, ACS Publications.

Faul et al. [94] synthesized the fluorescent microporous PI networks from the condensation of perylene-3,4,9,10-tetracarboxylic dianhydride (PTCDA) and 1,3,5-triazine-2,4,6-triamine using a Lewis acid catalyst zinc acetate/imidazole complex in the presence (PI-57a, Figure 38) and absence (PI-57b, Figure 38) of DMSO as weak solvent template. The THF dispersions of PI-57a and PI-57b displayed strong yellow–green fluorescence and stand in contrast to the weak red fluorescence of PTCDA (Figure 38b). Compared with the PTCDA monomer, the fluorescence intensities and the fluorescence quantum yield of PI-57 increased by 7 and 21 times (0.85 vs. 0.04), respectively. The authors found that the reduced π–π stacking of PI-57 relative to PTCDA would likely hinder self-quenching that arises from strong interactions among the perylene molecules and, hence, explains the amplified fluorescence. The successful construction of new microporous PI-57 networks and derived N-containing carbons is shown here to provide promising CO_2_ sorbents, while their fluorescent properties can be exploited for simple sensing of Fe^3+^.

**Figure 39:**
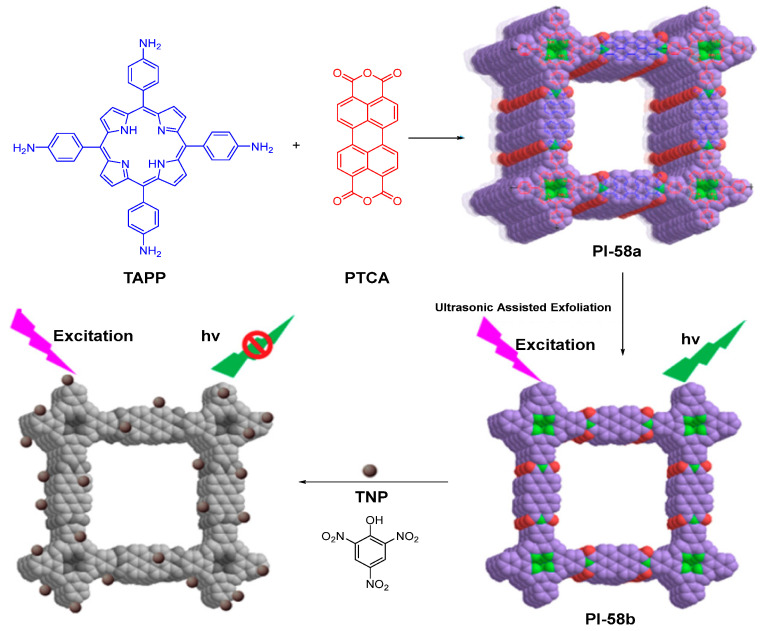
The structure of PI-58. Adapted with permission from Ref. [95]. Copyright 2017, ACS Publications.

Xian et al. [95] synthesized a fluorescent PI COF (PI-58a, Figure 39) through the solvothermal route using tetra(4-aminophenyl) porphyrin (TAPP) and perylenetracarboxylic dianhydride (PTCA). Due to the existence of a p–n heterojunction between TAPP and PTCA units in the PI-58a, the PI-58a shows a fluorescence at 500 nm with a relative *Φ*_PL_ value of 4%. Furthermore, few-layered PI covalent organic nanosheets (PI-58b) can be easily obtained from the PI-58a through a facile liquid phase exfoliation approach, and PI-58b exhibits a stronger fluorescence than that of PI-58a. The relative *Φ*_PL_ value of PI-58b is increased to 8%. PI-58a was exfoliated to be few layered nanosheets, minimizing the aggregation and maximizing the availability of electron density among the layers. In addition, donor–acceptor charge transfer among the PI-58b was extended. All of these could enhance the fluorescence intensity of PI-58b. Given the electron-rich benzene ring, electron-deficient imide structure, and good fluorescent properties, PI-58b has been successfully utilized as an efficient fluorescent probe for the highly sensitive and selective detection of 2,4,6-trinitrophenol (TNP).

**Figure 40:**
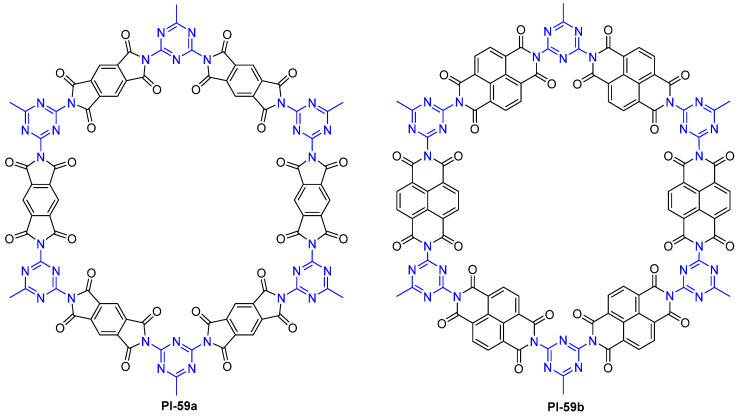
The structure of PI-59.

Yang et al. [96] developed two fluorescent PI-based porous COFs (PI-59, Figure 40) by directly heating the mixtures of melamine and PDMA and naphthalenetetracarboxylic dianhydride, respectively. PI-59a showed strong fluorescence in THF, DCM, and DMF solutions. The *Φ*_PL_ value of PI-59a was determined to be 41.6% with quinine sulfate as a reference. PI-59b emitted strong fluorescence in THF, acetonitrile, and DCM solutions. The *Φ*_PL_ value of PI-59b was determined to be 38.0%. These two PI COFs can emit strong fluorescence in appropriate solvents that originated from the π*–n transition caused by the high electrodelocalization and inherently rigid structure of the COF materials. The strong quenching effects of Fe^3+^ on the fluorescence of both PI COFs allowed selective sensing for Fe^3+^.

**Figure 41:**
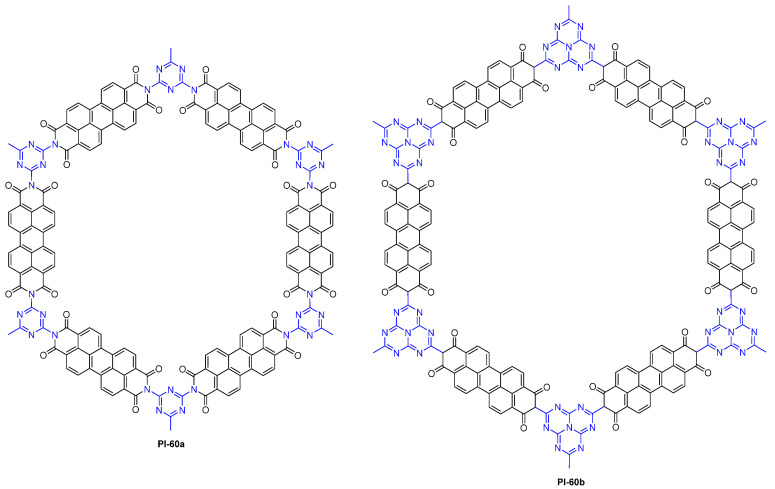
The structure of PI-60.

Wang et al. [97] constructed two crystalline PI COFs (PI-60, Figure 41) via a green method, in which no organic solvents were used. The authors found that both PI COFs with π-conjugated frameworks emitted strong fluorescence originating from the n–π* transition in DMF and the alkaline aqueous solution. More interestingly, both PI COFs showed obvious turn-off fluorescence performance for 10 metal ions (Fe^2+^, Cd^2+^, Co^2+^, Cr^2+^, Ni^2+^, Cu^2+^, Zn^2+^, Pb^2+^, Mn^2+^, and Fe^3+^) and 2 antibiotics (tetracycline and ofloxacin), which provides inspiration for the application of crystalline PI COFs with fluorescent and safety features in food detection fields.

**Figure 42:**
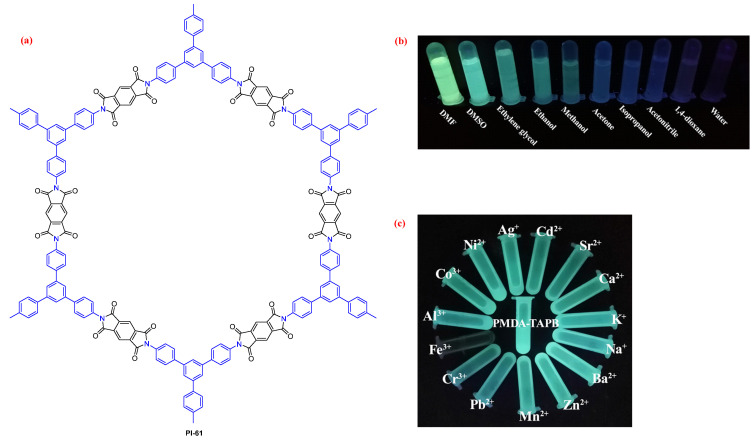
(**a**) The structure of PI-61. Photos of PI-61 in various solvents (**b**) and in DMF containing different metal ions (1 mM) (**c**) under 365 nm UV light. Adapted with permission from Ref. [98]. Copyright 2021, Elsevier.

Moreover, Zhao et al. [98] synthesized PI COFs (PI-61, Figure 42a) derived from PMDA and 1,3,5-tris(4-aminophenyl)benzene (TAPB). PI-61 exhibited a strong fluorescence in DMSO and DMF (Figure 42b). PI-61 also exhibited high sensitivity and selectivity to Fe^3+^ through fluorescence quenching (Figure 42c). The fluorescence quenching mechanism was revealed as the simultaneous effect of interaction between -CO- and Fe^3+^ and the absorption competition quenching (ACQ) mechanism.

**Figure 43:**
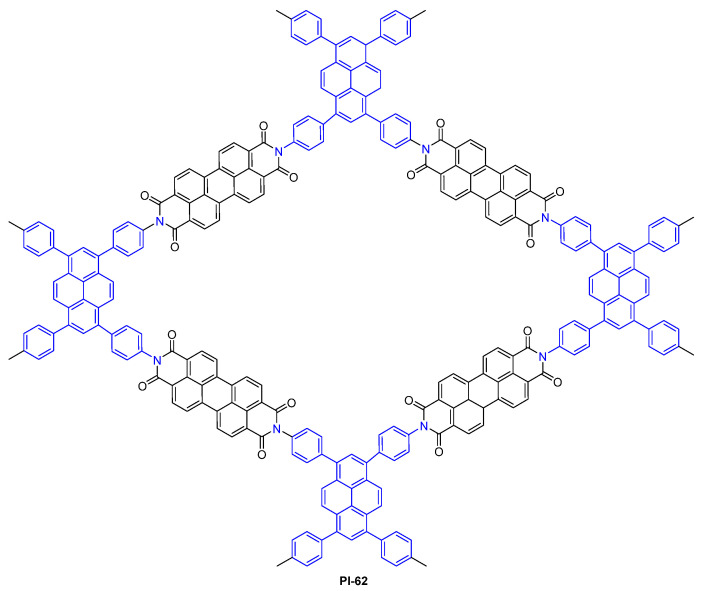
The structure of PI-62.

Abbaspourrad et al. [99] synthesized a fluorescent PI COF using perylene and pyrene building blocks (PI-62, Figure 43), via a one-pot condensation reaction. PI-62 emits a shiny blue luminescence under UV and visible light. This luminescence intensity is temperature-dependent in solvents with different polarities and dielectric constants, demonstrating that the PI-62 has potential use in a wide range of temperature-sensing devices. 

## 5. Conclusions and Outlook

On the one hand, it is possible to develop fluorescent aromatic PIs by suppressing CT interactions in PIs through appropriate molecular structure design. Compared with general fluorescent polymer materials, fluorescent aromatic PIs with outstanding overall properties show greater advantages and potential applications in the field of photoconducting devices. However, the inherent CT interactions in aromatic PIs inevitably cause fluorescence quenching. Therefore, the fluorescence quantum efficiency of aromatic PIs existing in the literature is relatively low. In the future, the design and synthesis of novel fluorescent aromatic PIs with high fluorescence quantum efficiency and outstanding overall properties are still to be investigated. On the other hand, it is also possible to develop high-efficiency fluorescent semiaromatic PIs, due to the suppression of CT interactions by introducing the alicyclic structure. However, the overall properties, especially the thermal properties, of fluorescent semiaromatic PIs are poorer than those of the aromatic PIs, which limits its wide application in the fields of photoconducting devices.

In summary, the general strategy to improve the fluorescence efficiency of PIs is summarized in this paper, which is of profound significance in guiding the design of new high-performance fluorescent PI materials. On this basis, the main research directions in the future are the following: obtaining key technologies of the controllable preparation of high-efficiency fluorescent PIs with outstanding overall properties; the regulation of the fluorescent color of PI materials; and carrying out this research in OLED applications.

## Data Availability

No new data were created.
